# Solvent Molecule-Induced Competitive Ion–Molecule Nucleophilic Substitution Reactions Involving an α‑Nucleophilic Reagent

**DOI:** 10.1021/acsomega.6c00428

**Published:** 2026-06-29

**Authors:** Gang Fu, Siwei Zhao, Hongyi Wang, Shiyue Liang, Jiaxu Zhang, Xiang bai, Jiabin Zhu, Bate Nasen, Qibin Liang, Li Yang

**Affiliations:** † Key Laboratory of Chemistry and Chemical Engineering on Heavy-Carbon Resources, School of Chemistry and Chemical Engineering, 172565 Yili Normal University, Yining 835000, P. R. China; ‡ School of Food Engineering, Harbin University, Harbin 150086, P. R. China; § MIIT Key Laboratory of Critical Materials Technology for New Energy Conversion and Storage, School of Chemistry and Chemical Engineering, 47822 Harbin Institute of Technology, Harbin 150001, P. R. China; ∥ Beijing Normal-Hong Kong Baptist University, Zhuhai 519087, P. R. China

## Abstract

Under microsolvent conditions, when the α-nucleophile HOO^–^ reacts with CH_3_Br, it can abstract a proton from the solvent CH_3_OH to induce alternative nucleophile CH_3_O^–^(HOOH). In the present work, the competition between the normal HOO^–^–S_N_2 pathway and the photon transfer (PT)-induced CH_3_O^–^–S_N_2 pathway has been explored by quantum chemistry calculations for HOO^–^(CH_3_OH)_*n*_ and CH_3_O^–^(HOOH)_0,1_ (CH_3_OH)_*n*−1_ reacting with CH_3_Br. The potential energy profile of the HOO^–^–S_N_2 pathway exhibits that the energy barriers of the traditional back-side attack substitution are 0.4–3.8 kcal/mol lower than those of the CH_3_O^–^–S_N_2 pathway, suggesting the HOO^–^(CH_3_OH)_*n*_ is more active, which is consistent with the experimental phenomenon. The activation strain analysis suggests compared to the CH_3_O^–^–S_N_2 pathway, the stronger interaction energy between the HOO^–^(CH_3_OH) and CH_3_Br over the entire reaction course stabilizes its transition state, which is caused by the stronger orbital interaction of the HOO^–^–S_N_2 pathway. The barrier heights of both pathways are increased with the incremental hydration, but the PT-induced CH_3_O^–^–S_N_2 pathway is highly suppressed. The enhanced reactivity of reactions involving HOO^–^(CH_3_OH)_*n*_ is found through comparison with CH_3_O^–^(HOOH)_0,1_(CH_3_OH)_*n*−1_ nucleophiles and is ascribed to the α-nucleophilic character of the HOO^–^ anion. This work deepens an understanding of the nature of the α-effect nucleophile and highlights the effect of the solvent molecule on the enhanced reactivity.

## Graphical abstract

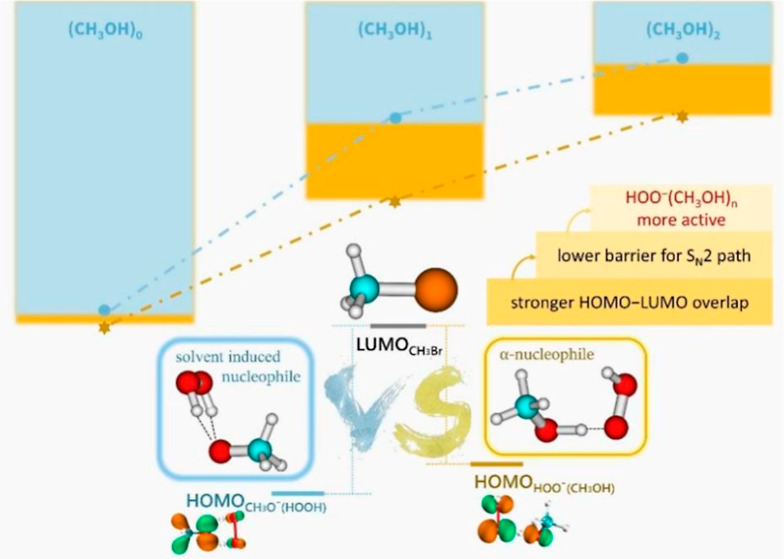

## 1 Introduction

The bimolecular nucleophilic substitution (S_N_2) reaction is a fundamental organic reaction and is widely studied from experimental and theoretical aspects.^1–8^ The S_N_2 reaction is found to be strongly affected by the solvent effect,^9,10^ and microsolvation provides a way to investigate reactions with a controlled number of solvent molecules.^11–14^ The experimental and computational studies on microsolvated S_N_2 reactions X^–^(HM) + CH_3_Y reactions, where HM is the protic solvent, have uncovered that the solvent molecules HM could alter the reactivity and reaction dynamics and even induce the new reaction pathways.^15–20^

The interaction between the nucleophile X^–^ and solvent HM (HM = H_2_O, NH_3_, and CH_3_OH)^20–23^ for X^–^(HM) + CH_3_Y reactions can form an alternative nucleophile M^–^(HX) by proton transfer, which could induce a new S_N_2 product channel, i.e., M^–^(HX) + CH_3_Y → CH_3_M + HX + Y^–^. Bierbaum et al.^20^ reported that the reactions of the microsolvated hydrogen peroxide anion, HOO^–^(CH_3_OH), with methyl halides resulted in the formation of the bare Y^–^ ion and the Y^–^(CH_3_OH) cluster, i.e., HOO^–^(CH_3_OH) + CH_3_Y → CH_3_OOH + Y^–^ + CH_3_OH (1a), without any Y^–^(HOOH) species following formula CH_3_O^–^(HOOH) + CH_3_Y → CH_3_OCH_3_ + Y^–^ + HOOH (1b). Although the Y^–^(HOOH) species cannot be observed, experiments cannot exclude the CH_3_O^–^(HOOH)–S_N_2 path, since mass spectrometry cannot distinguish these two pathways due to the same ionic product Y^–^ formed for reactions 1a and 1b. Similarly, the SIFT experiments on the reaction between HOO^–^(H_2_O) and CH_3_Cl also showed that the HOO^–^ was the active nucleophile.^22^

The direct dynamics simulations on the HOO^–^(H_2_O) + CH_3_Cl reaction^21^ revealed a new HO^–^–S_N_2 pathway induced by the solvent water molecule, besides the major HOO^–^–S_N_2 pathway. Usually, the basicity of α-nucleophilic X^–^(HM) is similar to that of the normal nucleophilic M^–^(HX), but the X^–^(HM)–S_N_2 path is much favorable than the M^–^(HX)–S_N_2. The reason for this intriguing feature still remains largely unclear. Besides, the interesting problems on how does α-nucleophilic X^–^(HM) enhance the reactivity and how do the increased solvent molecules influence chemical reactions thermodynamically are deserved to probe.

In this study, a series of microsolvated HOO^–^(CH_3_OH)_*n*_ + CH_3_Br (*n* = 1–2) (**na**) reactions and their possible solvent-induced reactions CH_3_O^–^(HOOH)_0,1_(CH_3_OH)_*n*−1_ + CH_3_Br (**nb**) reactions have been explored in detail by quantum chemical analysis, where **a** or **b** refers to HOO^–^ or CH_3_O^–^, and **n** is the number of CH_3_OH molecules.

$${HOO}^{-}{\left({CH}_{3}OH\right)}_{n}+{CH}_{3}Br\to {CH}_{3}OOH+{Br}^{-}+{nCH}_{3}OH\;\left(na\right)$$

$${CH}_{3}O^{-}{\left(HOOH\right)}_{0,1}{\left({CH}_{3}OH\right)}_{n-1}+{CH}_{3}Br\to {CH}_{3}{OCH}_{3}+{Br}^{-}+\left(0,1\right)HOOH+\left(n-1\right){CH}_{3}OH\;\left(nb\right)$$

The purpose of this study is 3-fold. (1) It is important to make clear why the proton transfer-induced species M^–^(XH) exist in HOO^–^(HM) + CH_3_Y reactions. (2) Reaction dynamics simulations show that the alternative S_N_2 path is usually kinetically unfavored in HOO^–^(sol) + CH_3_Y reactions,^24^ but the reason remains largely unclear. In the current work, we compare the PES features of HOO^–^ and CH_3_O^–^–S_N_2 pathways to explain the origin of the enhanced reactivity of α-nucleophiles HOO^–^(CH_3_OH)_*n*_ relative to the normal nucleophile CH_3_O^–^(HOOH) with similar basicity. (3) The α-effect has been observed in the reactions of single-solvated HOO^–^ anions with methyl halides.^20^ It is of interest to clarify the source-induced α-effect, i.e., the nature of α-effect nucleophile or the role of solvent through investigating the α-effect in the HOO^–^(CH_3_OH)_*n*_ + CH_3_Y reactions with increased number of solvent molecules.

### 1.1 Computational Methods

The HOO^–^(CH_3_OH)_*n*_ (**na**) and CH_3_O^–^(HOOH)_0,1_(CH_3_OH)_*n*−1_ (**nb**) + CH_3_Br are investigated at the MP2/ECP/d^25–27^ level of theory, which has been used in a previous study of the HOO^–^(H_2_O)_*n*_ + CH_3_Y (*X* = F, Cl, Br, I) and HOO^–^(NH_3_)_*n*_ + CH_3_Cl reactions and shows good performance.^21,28^ Based on the optimized geometries at the MP2/ECP/d level, the CCSD­(T) calculations^29^ coupled with the PP/t^30^ or PP/d^31^ basis set are performed to serve as the benchmark for the current reactions with or without solvent. Br and all other atoms are treated by the aug-cc-pVT­(D)­Z-PP and aug-cc-pVT­(D)­Z, respectively, which is defined as the PP/t (PP/d) basis set. The energetics in the solution phase has been computed using solvent methanol with the continuum solvation model PCM.^32^ The correct connection between transition state (TS) and entrance- or exit-channel complexes is confirmed by the intrinsic reaction coordinate (IRC) calculations.^33,34^ Gaussian 09 program is used for all calculations.^35^

## 2 Results and Discussion

### 2.1 Potential Energy Profile

The potential energy surfaces (PESs) of the HOO^–^(CH_3_OH)_*n*_ reacting with CH_3_Br reactions by using the MP2/ECP/d level of theory are shown in Figure S1 of the Supporting Information.^36^ As expected, a hydrogen-bond complex (0*a*/1a/2aRC1) and a traditional ion–dipole complex (0*a*/1a/2aRC2) are found for the entrance channel of the back-side attack substitution, and they can transfer to each other with a lower-energy-barrier transition state (0*a*/1a/2aTS_RC_). Subsequently, the system enters the Walden inversion transition state (0*a*/1a/2aTS_iS_), where the terminal oxygen atom of HOO^–^(CH_3_OH)_*n*_ attacks the carbon atom of CH_3_Br, while the C–Br bond elongates, causing the system to fall into a deep postreaction complex well. Except the back-side attack substitution pathway, a higher energy barrier front-side attack substitution (ret-S_N_2) pathway also exists in the HOO^–^(CH_3_OH)_*n*_ + CH_3_Br reactions, which is not competitive at room temperature, due to the repulsion of the crowed nucleophile and the leaving group. For exploring the variation of HOO^–^(CH_3_OH)_*n*_ + CH_3_Br reactions with the number of solvent molecule, the PES profiles of most favorable inv-S_N_2 channels are shown in Figure 1, together with the respective structures as shown in Figure 2.

**Figure 1:**
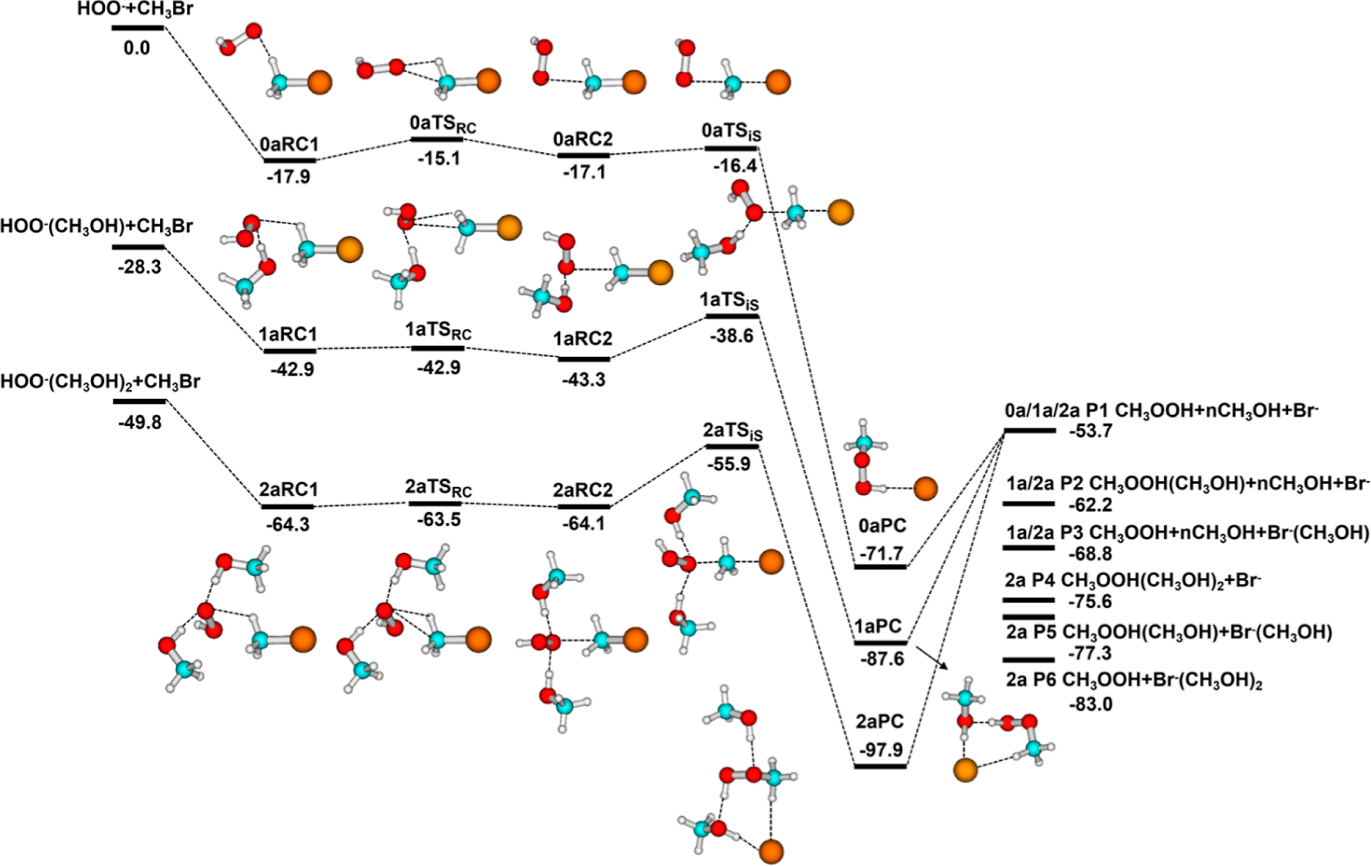
Potential energy surface of the HOO^–^(CH_3_OH)_*n*_ + CH_3_Br reactions displaying the back-side attack S_N_2 pathway by using the MP2/ECP/d method. Energy values (in kcal/mol) without ZPE.

**Figure 2:**
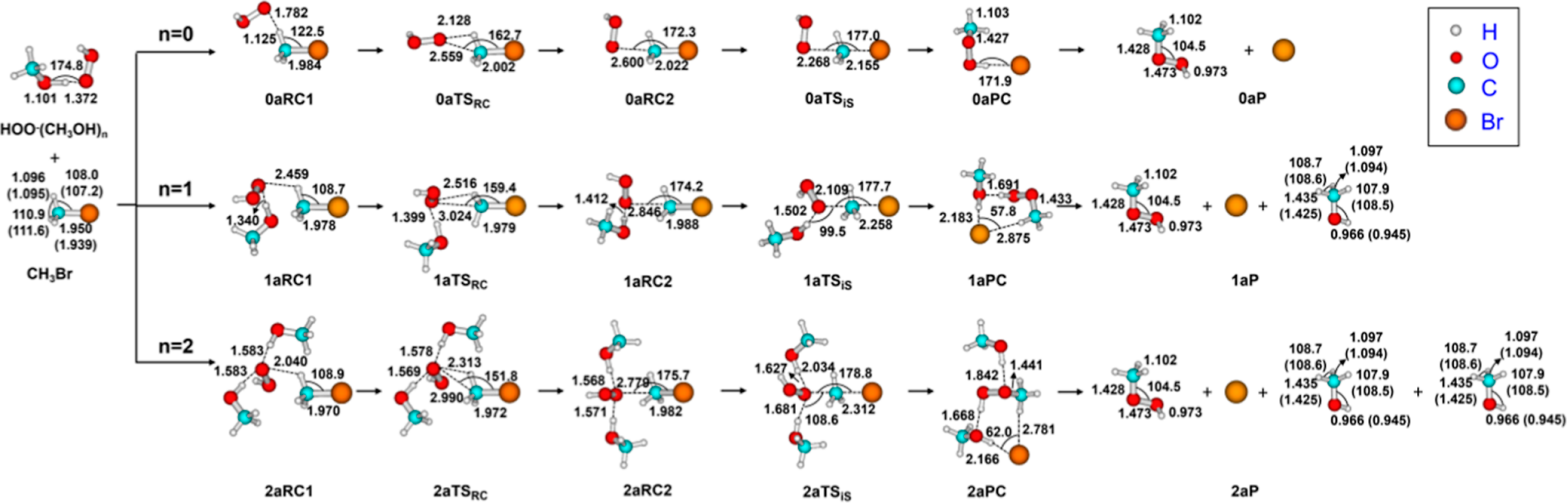
Structures of stationary points on the PESs of HOO^–^(CH_3_OH)_*n*_ + CH_3_Br reactions. Color code: H, white; C, cyan; O, red; Br, brown.

Since HOO^–^ is an active nucleophile, it can react with a solvent molecule to form an alternative nucleophile anion CH_3_O^–^(HOOH) by proton transfer from the CH_3_OH, which can serve as a nucleophile to induce a new S_N_2 pathway competing with HOO^–^(CH_3_OH)_*n*_, which is similar to the case of HOO^–^(H_2_O)_*n*_ converting to HO^–^(HOOH).^22,24,37^ The corresponding PES and geometrical structures of the inv-S_N_2 channel for CH_3_O^–^(HOOH)_0,1_(CH_3_OH)_*n*−1_ reacting with CH_3_Br at the MP2/ECP/d level of theory are shown in Figures 3 and 4, together with the unfavorable ret-S_N_2 pathway in Figure S2. As shown in Figure 3, the terminal oxygen in the nucleophilic reagent CH_3_O^–^(HOOH)_0,1_(CH_3_OH)_*n*−1_ forms a hydrogen-bonded complex nbRC1 with the hydrogen atom in CH_3_Br. Subsequently, the oxygen atom approaches the carbon in CH_3_Br, converting via the nbTS_RC_ transition state into an ionic dipole complex nbRC2. This then undergoes a Waldon inversion to yield the nucleophilic substitution product. The CH_3_O^–^–S_N_2 and HOO^–^–S_N_2 paths exhibit similar shapes on the potential energy surface diagram. It is of interest to compare with the PES features of HOO^–^– and CH_3_O^–^–S_N_2 paths to reveal which path is kinetically and thermodynamically favored.

**Figure 3:**
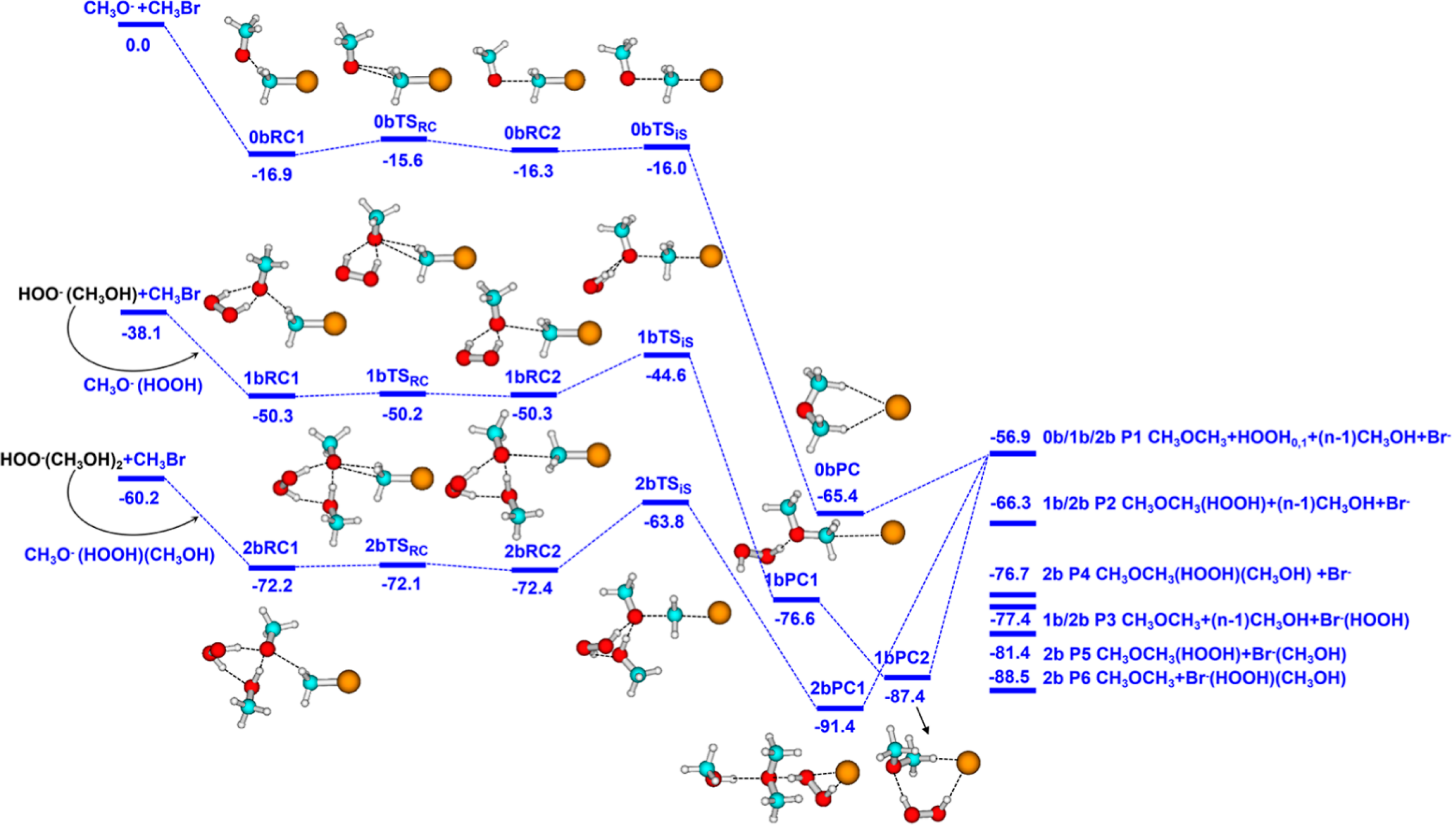
Potential energy surface of the CH_3_O^–^(HOOH)_0,1_(CH_3_OH)_*n*−1_ + CH_3_Br reactions displaying the back-side attack S_N_2 pathway by using the MP2/ECP/d method. Energy values (in kcal/mol) without ZPE. The CH_3_O^–^ + CH_3_Br reactants are set as the reference point.

**Figure 4:**
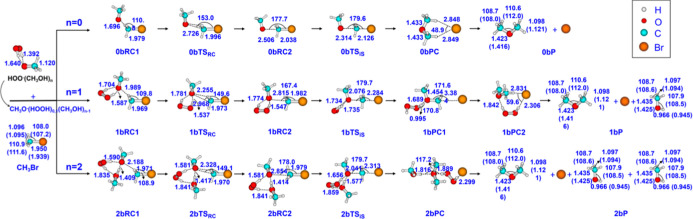
Structures of stationary points on the PESs of CH_3_O^–^(HOOH)_0,1_(CH_3_OH)_*n*−1_ + CH_3_Br reactions. Color code: H, white; C, cyan; O, red; Br, brown.

The stationary point energies for the HOO^–^(CH_3_OH)_*n*_ and CH_3_O^–^(HOOH)_0,1_(CH_3_OH)_*n*−1_ + CH_3_Br reactions calculated at MP2/ECP/d levels are compared with the available experimental values^37^ and CCSD­(T)/PP/t­(d) benchmark as summarized in Table 1. The experimental reaction exothermicities with ZPE at 0 K are −50.7 and −54.9 kcal/mol for 0aP and 0bP, respectively.^37^ The MP2 method gives a good agreement with the experiment, which differs by 0.4–2.3 kcal/mol. For all of the stationary points, the largest difference is less than 3.5 kcal/mol between MP2 and CCSD­(T) energies, suggesting that the MP2 method is reliable for the current study and the following discussions.

**Table 1:** Electronic Structure Theory Energies for Stationary Points of the S_N_2 Pathway on the PES of HOO^–^(CH_3_OH)_*n*_/CH_3_O^–^(HOOH)_0,1_(CH_3_OH)_*n*−1_ + CH_3_Br (*n* = 0–2) Reactions with Different Methods^a^ ^b^

species	MP2	CCSD(T)	species	MP2	CCSD(T)
HOO^–^(CH_3_OH)_*n*_ + CH_3_Br			CH_3_O^–^(HOOH)_0,1_(CH_3_OH)_*n*−1_ + CH_3_Br		
HOO^–^	0.0	0.0	CH_3_O^–^	0.0	0.0
0aRC1	–17.9	–17.1	0bRC1	–16.9	–16.5
0aTS_RC_	–15.1	–14.7	0bTS_RC_	–15.6	–15.4
0aRC2	–17.1	–16.9	0bRC2	–16.3	–16.1
0aTS_iS_	–16.4	–16.5	0bTS_iS_	–16.0	–16.0
0aTS_rS_	14.4	12.7	0bTS_rS_	18.9	18.4
0aPC	–71.7	–69.2	0bPC	–65.4	–64.5
0aP	–53.7 (−51.1)	–50.4 (−47.8) [–50.7]	0bP	–56.9 (−52.6)	–56.0 (−51.7) [–54.9]
HOO^–^(CH_3_OH)	0.0	0.0	CH_3_O^–^(HOOH)	0.0	0.0
1aRC1	–14.7	–14.9	1bRC1	–12.2	–12.3
1aTS_RC_	–14.6	–14.9	1bTS_RC_	–12.1	–12.1
1aRC2	–15.0	–15.3	1bRC2	–12.1	–12.4
1aTS_iS_	–10.3	–11.9	1bTS_iS_	–6.5	–8.0
1aTS_rS_	22.5	19.0	1bTS_rS_	29.7	26.4
1aPC	–59.3	–59.1	1bPC1	–38.4	–39.1
			1bTS_PC_	–38.2	–38.9
			1bPC2	–49.3	–50.6
1aP1	–25.5	–25.6	1bP1	–18.8	–20.0
1aP2	–33.9	–34.0	1bP2	–28.1	–29.3
1aP3	–40.6	–40.3	1bP3	–39.3	–40.3
HOO^–^(CH_3_OH)_2_	0.0	0.0	CH_3_O^–^(HOOH)(CH_3_OH)	0.0	0.0
2aRC1	–14.5	–14.8	2bRC1	–12.0	–12.2
2aTS_RC_	–13.7	–14.0	2bTS_RC_	–11.9	–12.1
2aRC2	–14.4	–14.6	2bRC2	–12.2	–12.4
2aTS_iS_	–6.1	–7.6	2bTS_iS_	–3.6	–5.4
2aTS_rS_	25.7	23.2	2bTS_rS_	34.8	31.1
2aPC	–48.1	–47.7	2bPC	–37.6	–39.2
2aP1	–4.0	–3.9	2bP1	3.3	1.9
2aP2	–12.4	–12.4	2bP2	–6.1	–7.3
2aP3	–19.1	–18.7	2bP3	–17.2	–18.3
2aP4	–25.8	–25.5	2bP4	–16.5	–17.6
2aP5	–27.5	–27.1	2bP5	–21.2	–22.0
2aP6	–33.2	–33.3	2bP6	–28.3	–29.5

a The numbers in parentheses and brackets denote calculated reaction energies with ZPE included and experimental values, respectively.

b CCSD­(T) combined with the PP/t and PP/d basis set was performed based on MP2/ECP/d optimized geometries, serving as the benchmark for the gas and microsolvated case, respectively.

### 2.2 Competition between HOO^–^–S_N_2 and CH_3_O^–^–S_N_2 Pathways

#### 2.2.1 Microsolvated Nucleophiles and Reaction Enthalpies

The theoretical studies of HOO^–^(HM) + CH_3_Y reactions^20^ pointed out the nucleophile tended to form an alternative nucleophile by transferring a proton from the solvent molecule. To figure out the reason, the color-filled map of electron location function (ELF) generated by Multiwfn program^38,39^ is presented in Figure 5, where the blue-to-red region indicates low-to-high ELF value. The ELF plot is between ranges 0.0 and 1.0, corresponding to the least to the greatest Pauli repulsion. The results indicate that the electrons are well delocalized or localized.^40–42^ It can be seen that HOO^–^(CH_3_OH) gives a higher ELF value with a cyan color between the two moieties (Figure 5a, as showed by arrows) relative to that observed for CH_3_O^–^(HOOH) (Figure 5b), indicating the stronger interactions between HOO^–^ and CH_3_OH. Thus, HOO^–^ has a greater tendency to abstract the hydrogen of the methanol molecule to form an alternative nucleophile CH_3_O^–^(HOOH). This result is consistent with the relative energy as shown in Table S1 that for the single-solvated species, CH_3_O^–^(HOOH) (**1b**) is 4 kcal/mol lower in energy than the HOO^–^(CH_3_OH) (**1a**). Introducing a second CH_3_OH molecule to the HOO^–^ anion, the blue area surrounding the HOO^–^/CH_3_O^–^ and solvent molecules indicates the very low electron localization zones with a low ELF value (Figure 5c,d, as showed by arrows), indicating the weak interaction between them. Generally, the CH_3_O^–^(HOOH)­(CH_3_OH)_*n*−1_ structures are thermodynamically more stable than HOO^–^(CH_3_OH)_*n*_ structures, which is similar to HOO^–^(H_2_O)_*n*_ + CH_3_Cl reactions,^28^ but Bierbaum’s group^20^ reported that an enhanced reactivity of HOO^–^(CH_3_OH) toward both methyl halides was observed as opposed to the normal nucleophiles CH_3_O^–^(HOOH).

**Figure 5:**
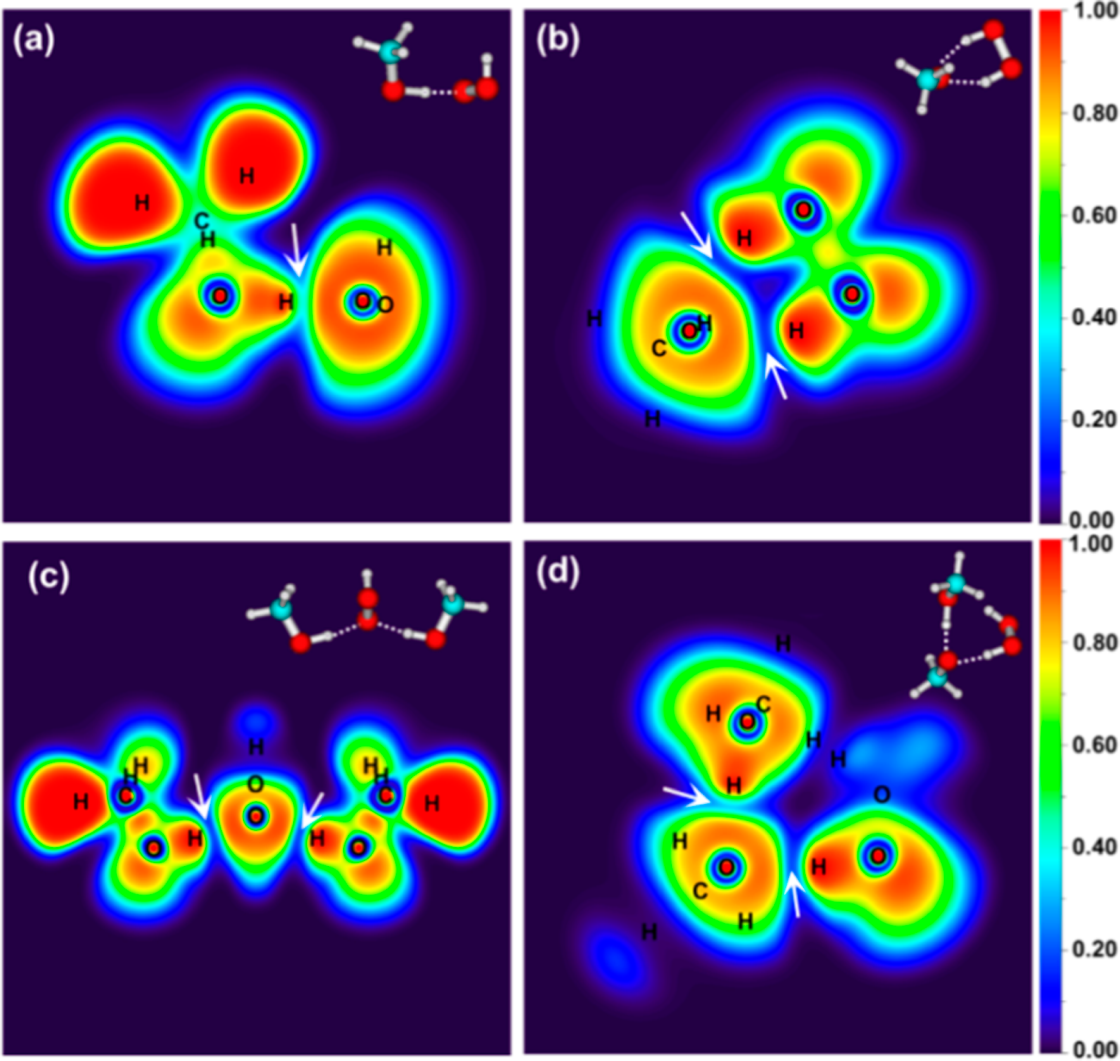
Color-filled map of ELF for (a) HOO^–^(CH_3_OH), (b) CH_3_O^–^(HOOH), (c) HOO^–^(CH_3_OH)_2_, and (d) CH_3_O^–^(HOOH)­(CH_3_OH).

The reaction enthalpy (Δ*H*) of the unsolvated HOO^–^ + CH_3_Br S_N_2 reaction (−53.7 kcal/mol) is less exothermic than that of the CH_3_O^–^ + CH_3_Br S_N_2 reaction (−56.9 kcal/mol), as listed in Table 1. The Δ*H* of **na**/**nb** (*n* = 1, 2) shows that the reaction becomes less exothermic with the addition of the first and second solvent molecules. In contrast to the unsolvated reactions, with the increased number of solvent molecules, the HOO^–^–S_N_2 paths (Δ*H* = −25.5/–4.0 kcal/mol) are more exothermic than their respective CH_3_O^–^–S_N_2 paths (Δ*H* = −18.8/3.3 kcal/mol) for single/double solvent molecules as shown in Table 1. This may contribute to the competitiveness of solvated HOO^–^–S_N_2 pathways relative to CH_3_O^–^–S_N_2 pathways. However, the most decisive factor that influences the reaction activity is the barrier height as discussed below.

#### 2.2.2 Overall Barrier Height

The overall barriers (Δ*E* ^‡^) defined as the TS and reactants energy difference for both HOO^–^–S_N_2 and CH_3_O^–^–S_N_2 reactions are listed in Table 1. Although the Δ*E* ^‡^ of both S_N_2 reaction pathways is increased as the degree of solvation increased, the Δ*E* ^‡^ of HOO^–^–S_N_2 (−16.4, −10.3, and −6.1 kcal/mol) is generally lower than that of CH_3_O^–^–S_N_2 (−16.0, −6.5, and −3.6 kcal/mol) reactions for *n* = 0, 1, and 2. This supports that HOO^–^–S_N_2 is more competitive than CH_3_O^–^–S_N_2, but the reason is deserved to be explored further. The relationship between the overall barrier and the HOMO energy of nucleophiles, charge distribution, and geometric character of TSs will be helpful for revealing the enhanced reactivity of HOO^–^(CH_3_OH)_*n*_ compared to the normal nucleophiles CH_3_O^–^(HOOH)­(CH_3_OH)_*n*−1_.

#### 2.2.3 HOMO Levels of Nucleophiles

The interaction between the HOMO of the nucleophile and the LUMO of substrate can be used to understand the competition between the HOO^–^– and CH_3_O^–^–S_N_2 path with solvation. As shown in Figures 6a and 7, the HOMO level of both HOO^–^(CH_3_OH)_1,2_ and CH_3_O^–^(HOOH)­(CH_3_OH)_0,1_ decreases with the increased number of solvent molecules of the nucleophile, resulting in the enlarged HOMO–LUMO gap. This also causes the weakened HOMO–LUMO interaction, destabilized TS, and raised Δ*E* ^‡^ of the solvated reactions. Bickelhaupt and Xie et al. also observed the similar negative correlation between the HOMO level of the microsolvated anionic nucleophile and barrier heights.^28,43^ As shown in Figure 6a, the overall barrier heights and HOMO energy level of nucleophiles show a good correlation, with the coefficient of determination *r* ^2^ = 0.99 for HOO^–^–S_N_2 and CH_3_O^–^–S_N_2 paths. In addition, the HOMO energy of HOO^–^ increases faster than that of CH_3_O^–^ with incremental solvation, which is in accordance with the faster decrease of the barrier heights for the HOO^–^–S_N_2 path than for the CH_3_O^–^–S_N_2 path. Therefore, the differential competition between HOO^–^– and CH_3_O^–^–S_N_2 pathways in the experiment can be interpreted by the HOMO–LUMO interaction model.

**Figure 6:**
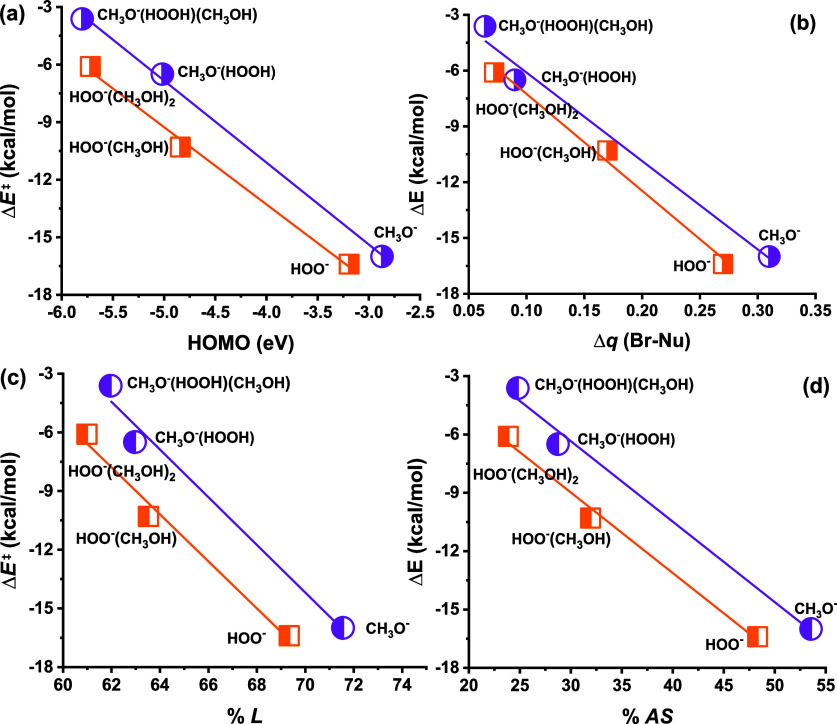
Correlations between barrier heights and the HOMO level of nucleophiles (a), variation of charge distribution (Δ*q*) (b), geometry looseness (c), and geometry asymmetry (d) for HOO^–^(CH_3_OH)_*n*_ + CH_3_Br and CH_3_O^–^(HOOH)_0,1_(CH_3_OH)_*n*−1_ + CH_3_Br reactions.

**Figure 7:**
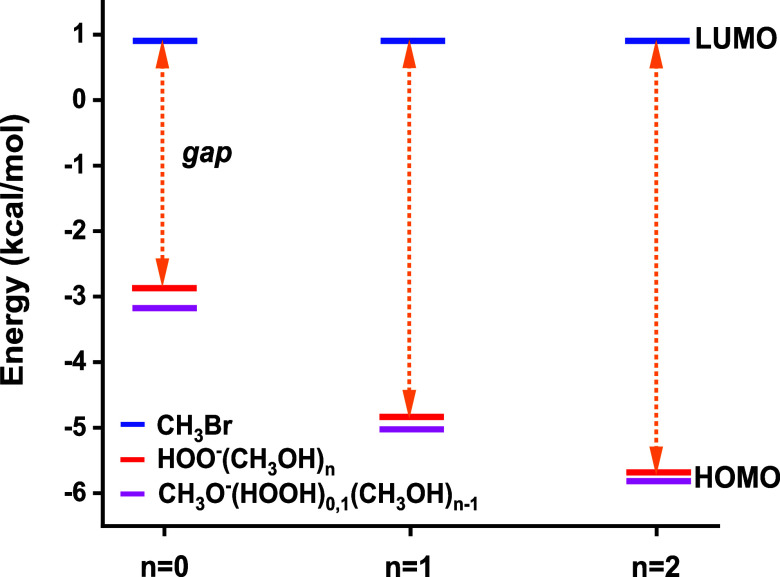
Energy level of the HOMO and LUMO for reactants.

#### 2.2.4 Charge Distribution

The atomic dipole moment-corrected Hirshfeld (ADCH) charge^44,45^ analysis in the Multiwfn program^38,39^ has been used to evaluate the asymmetry of the charge distributions for TSs, which is defined as Δ*q*(Br – Nu) = *q*(Br) – *q*(Nu) between the nucleophile and leaving group Br (see Table S2). A larger value of Δ*q*(Br – Nu) means less electrons transfer from the nucleophile to the leaving group and the TS is more product-like. Figure 6b presents that the Δ*q*(Br – Nu) value is slightly reduced with the incremental solvation, which is in line with the increased barrier heights with *r* ^2^ = 0.97–0.98 for both HOO^–^–S_N_2 and CH_3_O^–^–S_N_2 pathways. As shown in Figure 6b, in terms of the charge distribution, the Δ*q*(Br – Nu) of CH_3_O^–^ nucleophiles decreases quickly, and the calculated overall barrier for the CH_3_O^–^–S_N_2 path increases faster than that of the HOO^–^–S_N_2 path with incremental solvation. Therefore, the Δ*q*(Br – Nu) can also explain the difference of the barrier between HOO^–^ and CH_3_O^–^–S_N_2 pathways with the stepwise addition of the solvent molecules.

#### 2.2.5 TS Structure

The shortening of the O–C bond and the elongation of the C–Br bond for TS of HOO^–^– and CH_3_O^–^–S_N_2 paths as the increased solvation are displayed in Figures 2 and 4. The TS structure looseness (%L) and geometrical asymmetry (%AS) can be used to evaluate variation of the TSs’ geometry for the HOO^–^(CH_3_OH)_*n*_ + CH_3_Br and CH_3_O^–^(HOOH)_0,1_(CH_3_OH)_*n*−1_ + CH_3_Br reactions.^28^

1a %L=%(O−C)+%(C−Br)

1b $$\%\left(C-Br\right)=100\times \left[r^{TS}\left(C-Br\right)-r^{Reactant}\left(C-Br\right)\right]/r^{Reactant}\left(C-Br\right)$$

1c $$\%\left(O-C\right)=100\times \left[r^{TS}\left(O-C\right)-r^{Product}\left(O-C\right)\right]/r^{Product}\left(O-C\right)$$

1d %AS=%(O−C)−%(C−Br)

where *r* ^TS^(C–Br) and *r* ^TS^(O–C) present the C–Br and O–C bond lengths of TS, respectively, *r* ^Reactant^(C–Br) presents the C–Br bond length of reactant CH_3_Br, and *r* ^Product^(O–C) presents the O–C bond length of the product CH_3_OOH or CH_3_OCH_3_. The corresponding values of %L and %AS are collected in Table S3, and their plots versus the overall barrier for the HOO^–^–S_N_2 and CH_3_O^–^–S_N_2 paths are shown in Figure 6c,d, which show good correlations (*r* ^2^ = 0.97–0.98). Obviously, the structures of TSs become tighter and more product-like under the role of microsolvation, resulting in the raised barrier heights. As shown in Figure 6c,d, the %L and %AS of CH_3_O^–^ nucleophiles drop faster than that of HOO^–^ nucleophiles, as n increases from 0 to 2, which is associated with the higher reaction barriers for the CH_3_O^–^–S_N_2 path than for the HOO^–^–S_N_2 path. Noncovalent interaction images also support this conclusion. With the introduction of solvent molecules, more regions of weak interactions formed with CH_3_O^–^ (green and blue areas in Figure S3), indicating that both the CH_3_O^–^ and CH_3_ fragments were better solvated. This resulted in a more compact transition state structure, ultimately leading to decreased reactivity.

#### 2.2.6 Energy Decomposition Analysis (EDA) and Frontier Molecular Orbital

To explain the experimental phenomenon, we performed energy decomposition analysis for the HOO^–^(CH_3_OH) + CH_3_Br and CH_3_O^–^(HOOH) + CH_3_Br reactions. The energy difference between TSs and reactants, Δ*E*, can be decomposed to preparation energy (Δ*E*_prep_) and interaction energy (Δ*E*_int_) as described elsewhere:^46^

$$\Delta E=\Delta E_{prep}+\Delta E_{\mathrm{int}}$$

As shown in Figure 8, the preparation energy is hardly affected by varying the nucleophiles, whereas CH_3_O^–^(HOOH) manifests in destabilizing the interaction energy, which further results in a higher S_N_2 reaction barrier. Thus, the nucleophile HOO^–^(CH_3_OH) reacts with CH_3_Br more favorably than the CH_3_O^–^(HOOH). The key occupied–unoccupied orbital interaction between the 2p atomic orbital of HOO^–^(CH_3_OH) and CH_3_O^–^(HOOH), i.e., HOMO_HOO–(CH3OH)_ and HOMO_CH3O–(HOOH)_, and the antibonding σ*_C–Br_ unoccupied orbital of CH_3_Br (LUMO_CH3Br_) has been analyzed. As shown in Figure 9, a smaller HOMO_HOO–(CH3OH)_–LUMO_CH3Br_ energy gap caused the stronger orbital interaction for the HOO^–^(CH_3_OH)–S_N_2 pathway, compared to the CH_3_O^–^(HOOH)–S_N_2 pathway. In brief, a more stabilized HOMO of CH_3_O^–^(HOOH) compared to that of the HOO^–^(CH_3_OH) leads to a larger HOMO–LUMO_CH3Br_ gap and thus causes the lower reactivity with a very high energy barrier.

**Figure 8:**
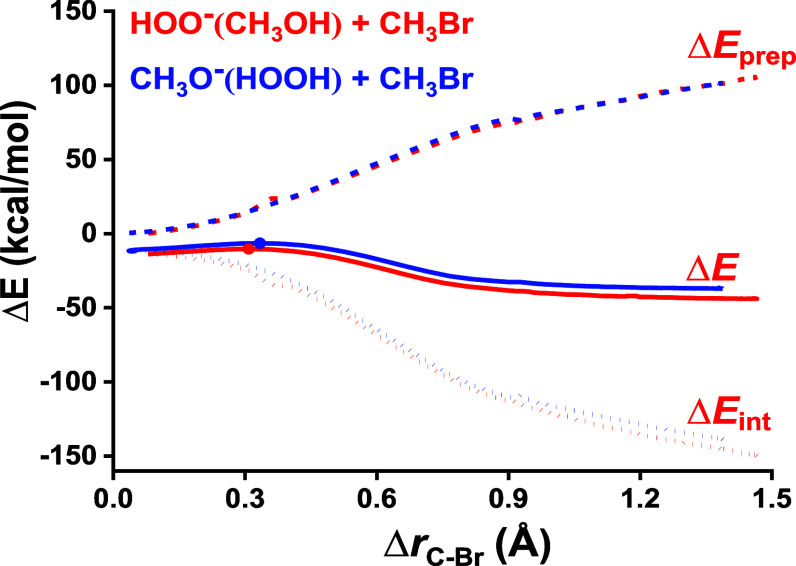
Activation strain analysis of HOO^–^(CH_3_OH) + CH_3_Br and CH_3_O^–^(HOOH) + CH_3_Br reactions.

**Figure 9:**
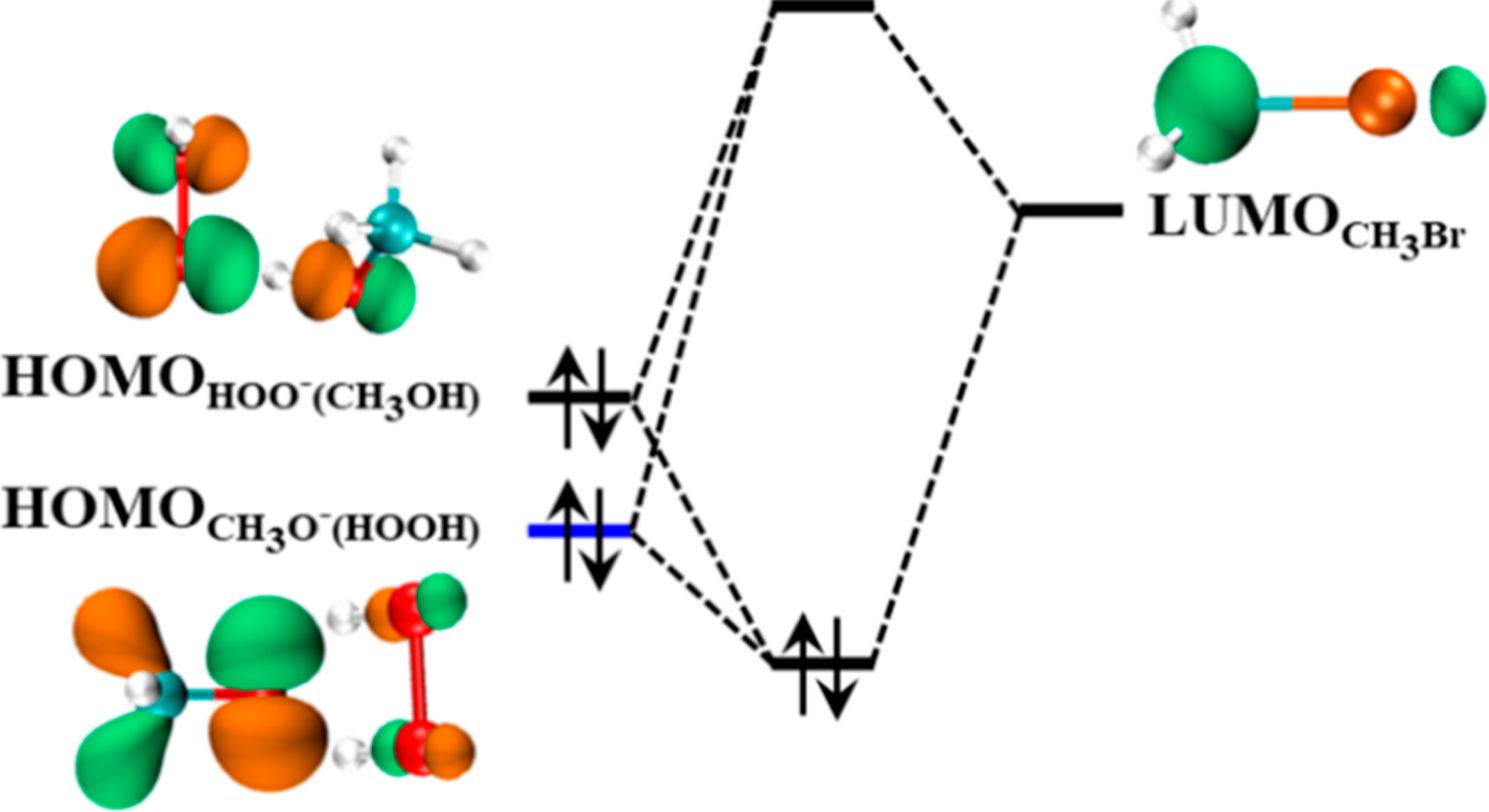
Schematic representation of the frontier molecular orbital interactions of the most important HOMO–LUMO interaction.

### 2.3 Microsolvation Effect

#### 2.3.1 PCM Model

The effects of the individual solvent molecules on the competition between HOO^–^–S_N_2 and CH_3_O^–^–S_N_2 paths have been analyzed through comparing the barrier heights of the microsolvated system in the gas phase and the liquid phase using a PCM implicit methanol model. As depicted in Figure 10, the overall barrier heights of both HOO^–^–S_N_2 and CH_3_O^–^–S_N_2 pathways increase with incremental hydration under both the microsolvated and bulk solvation conditions, but for the latter, they increase at a lower rate. This can be reflected by the HOMO level change of the nucleophiles. As shown in Table S4, the HOMO levels of HOO^–^ with or without CH_3_OH are similar, so it is estimated that the barrier height under bulk solvation may soon reach a plateau. The barrier difference between CH_3_O^–^– and HOO^–^–S_N_2 path is 0.4 kcal/mol under unsolvated conditions, indicating that their competitive properties are similar and difficult to distinguish. The increased degree of solvation enlarges the barrier difference with the value of 3.8 (*n* = 1), 2.5 (*n* = 2), and 1.1 (liquid phase) kcal/mol, respectively. And the HOO^–^–S_N_2 path becomes preferred compared to the CH_3_O^–^–S_N_2 path with the addition of methanol molecules.

**Figure 10:**
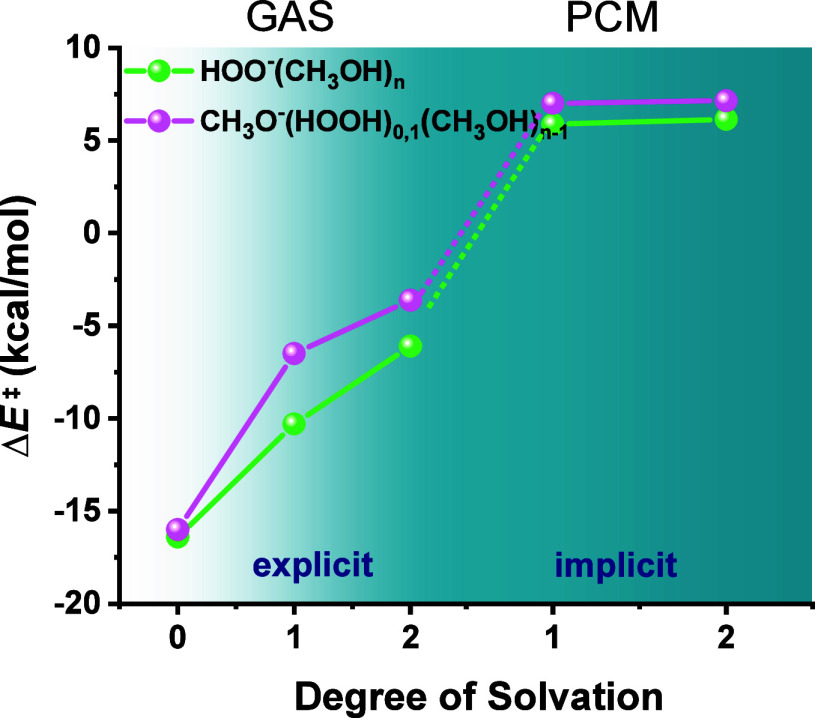
Barrier heights of inv-S_N_2 transition states as a function of degree of solvation n.

#### 2.3.2 α-Effect

The term α-effect^47^ proposed by Pearson and Edwards denotes a downward deviation from the Brønsted-type correlation of the normal nucleophiles. Here, the comparison between HOO^–^(CH_3_OH)_*n*_ and CH_3_O^–^(HOOH)_0,1_(CH_3_OH)_*n*−1_ suggests an existing α-effect. As shown in Figure 11, the barrier height of HOO^–^(CH_3_OH)_*n*_ deviates downward from the Brønsted-type reactivity–basicity relationship given by classical normal nucleophile CH_3_O^–^(HOOH)_0,1_(CH_3_OH)_*n*−1_, F^–^(CH_3_OH)_*n*_, and Cl^–^(CH_3_OH)_*n*_. The introduction of solvent molecules further amplifies this trend. The proton transfer-induced species CH_3_O^–^(HOOH) is higher in energy than HOO^–^(CH_3_OH), suggesting the enhanced reactivity of HOO^–^(CH_3_OH) ascribed to the α-nucleophilic character of the HOO^–^ anion.

**Figure 11:**
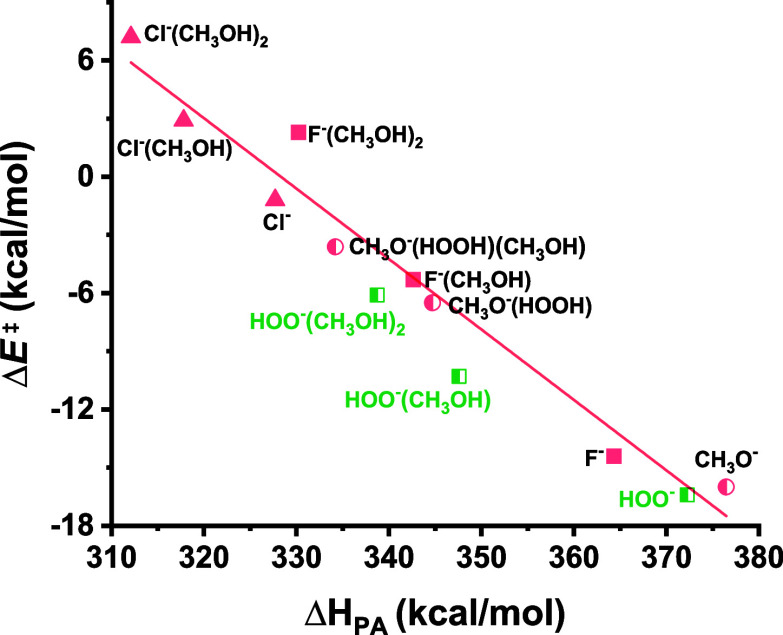
Correlations between proton affinity (PA) and barrier heights ΔE for HOO^–^(CH_3_OH)_*n*_ + CH_3_Br, CH_3_O^–^(HOOH)_0,1_(CH_3_OH)_*n*−1_ + CH_3_Br, F^–^(CH_3_OH)_*n*_ + CH_3_Br, and Cl^–^(CH_3_OH)_*n*_ + CH_3_Br reactions.

## 3 Conclusion

In this work, the PES profiles of the HOO^–^(CH_3_OH)_*n*_ + CH_3_Br reactions have been characterized by the CCSD­(T)/PP/t­(d)//MP2/ECP/d method. For the microsolvated nucleophile HOO^–^(CH_3_OH)_*n*_, proton transfer from CH_3_OH can induce the CH_3_O^–^(HOOH)_0,1_(CH_3_OH)_*n*−1_ nucleophile and the occurrence of the CH_3_O^–^–S_N_2 pathway. This is attributed to the stronger interactions between HOO^–^ and CH_3_OH, with a higher ELF value between the two moieties.

As the number of solvent CH_3_OH molecules increased, the barrier heights of the HOO^–^–S_N_2 pathway increased and those for the CH_3_O^–^–S_N_2 path increased slightly faster. The TS of CH_3_O^–^–S_N_2 is 0.4–3.8 kcal/mol higher than that of HOO^–^–S_N_2, suggesting the latter path is thermodynamically favored. However, the relatively small energy difference makes it only a qualitative prediction. A quantitative competitive relationship requires more precise dynamic simulations to provide. The HOMO level of nucleophiles and barrier heights shows a good correlation. The higher HOMO level of the HOO^–^ nucleophiles induced by solvent molecules is found when compared to that of CH_3_O^–^ nucleophiles, resulting in a stronger HOMO–LUMO overlap with the substrate and a lower barrier for the HOO^–^–S_N_2 path.

It is found that the TS structures of the CH_3_O^–^–S_N_2 path are tighter and more product-like than the HOO^–^–S_N_2 path with the incremental solvation. The activation strain analysis indicates the more unstabilizing transition states of the CH_3_O^–^–S_N_2 path contributing to the higher barrier. The barrier of HOO^–^(CH_3_OH)_*n*_ deviates downward from the Brønsted-type reactivity–basicity relationship line as given by normal nucleophile CH_3_O^–^(HOOH)_0,1_(CH_3_OH)_*n*−1_, suggesting that the reactions with α-nucleophile HOO^–^(CH_3_OH)_*n*_ would show an enhanced reactivity. Further dynamics simulation is desired and ongoing to explore the possible dynamical factors on the α-effect reactions.

## Supplementary Material



## Notes

Energy values, geometries, and other properties of the stable points on the potential energy surface for the reactions of HOO^–^(CH_3_OH)_*n*_ and CH_3_O^–^(HOOH)_0,1_(CH_3_OH)_*n*−1_ + CH_3_Br (PDF)
