# Supplementary material for: Solvent Molecule-Induced Competitive Ion–Molecule Nucleophilic Substitution Reactions Involving an α‑Nucleophilic Reagent
Source: ACS Omega. 2026 Jun 29;11(27):39831–40. doi: 10.1021/acsomega.6c00428 (PMC13382678; doi:10.1021/acsomega.6c00428)
Supplement: Supplementary file 1 [file ao6c00428_si_001.pdf]

**Solvent molecule induced competitive ion-molecule nucleophilic substitution reactions involving α-nucleophilic reagent**

Gang Fu,[a,#] Siwei Zhao,[b,#] Hongyi Wang,[c] Shiyue Liang,[d] Jiaxu Zhang,[a,c,*] Xiang bai,[a,*] Jiabin Zhu,[a] Bate Nasen,[a] Qibin Liang,[a] and Li Yang[a,*]

[a] Key Laboratory of Chemistry and Chemical Engineering on Heavy-Carbon Resources, School of Chemistry and Chemical Engineering, Yili Normal University, Yining 835000, P. R. China

[b] School of Food Engineering, Harbin University, Harbin 150086, P. R. China

[c] MIIT Key Laboratory of Critical Materials Technology for New Energy Conversion and Storage, School of Chemistry and Chemical Engineering, Harbin Institute of Technology, Harbin 150001, P. R. China

[d] Beijing Normal-Hong Kong Baptist University, Zhuhai 519087, P. R. China

Author E-mail Address: baixianglnu@163.com, yangli@ylnu.edu.cn and zhjx@hit.edu.cn

[#]Gang Fu and Siwei Zhao contributed equally to this work.

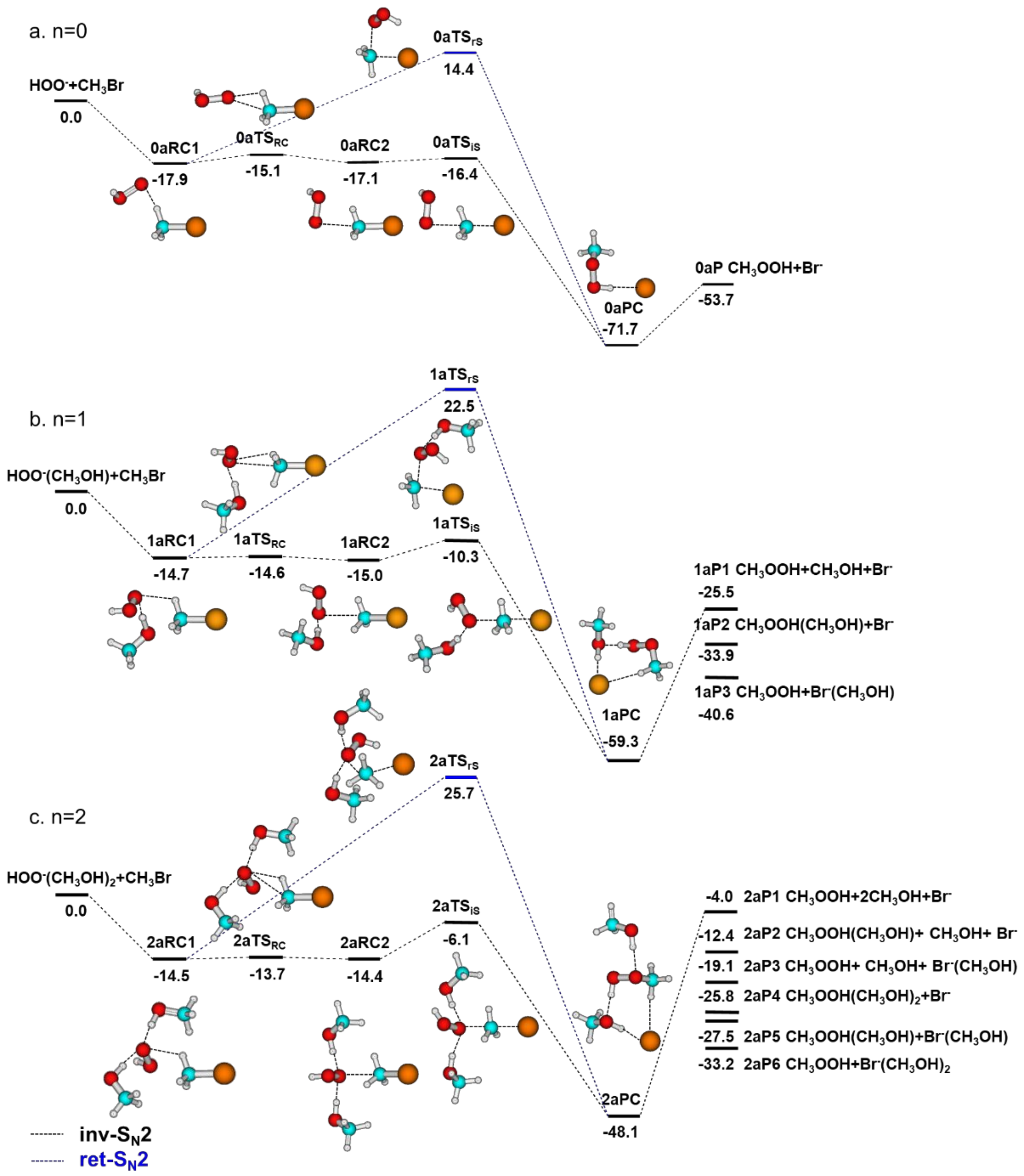


Figure S1. Potential energy profile for the $HOO^{-}$ $(CH_3OH)_n$ + $CH_3Br$ reactions with the MP2/ECP/d method.

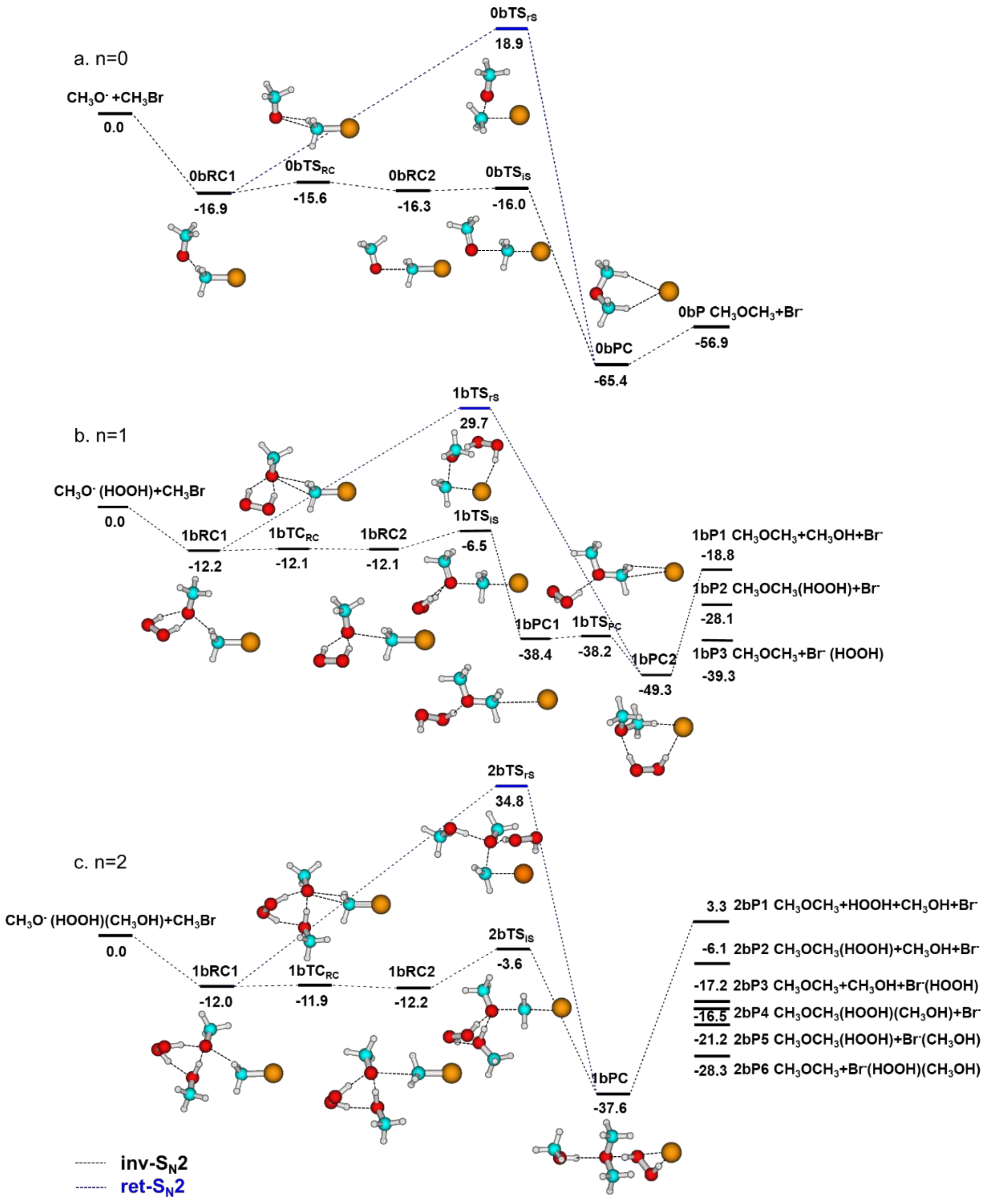


Figure S2. Potential energy profile for the $CH_3O^-(HOOH)_{0,1}(CH_3OH)_{n-1}$ + $CH_3Br$ reaction with the MP2/ECP/d method.

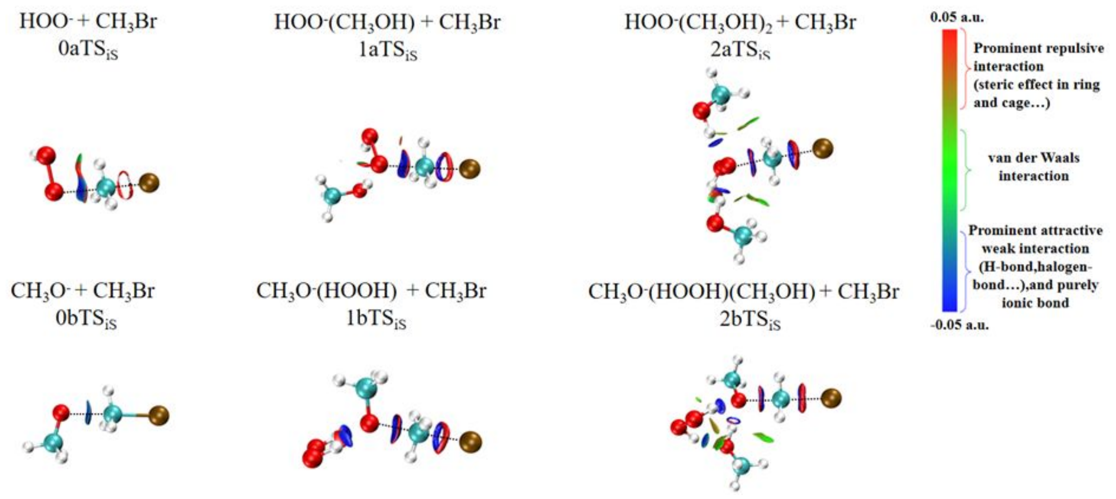


Figure S3. Visualization of noncovalent interactions (NCIs) in the inv-$S_N2$ transition state structures of $CH_3O^-(HOOH)_{0,1}(CH_3OH)_{n-1}$ + $CH_3Br$ reaction using the MP2/ECP/d method. Color codes: Cyan, White, Red and Ochre respectively represent C, H, O, and Br elements.

Table S1. Electronic structure theory energies for reactants of the $HOO^{-}(CH_3OH)_n$ + $CH_3Br$ and $CH_3O^{-}(HOOH)_{0,1}(CH_3OH)_{n-1}$ + $CH_3Br$ reactions with MP2/aug-cc-pVDZ method, and the $HOO^{-}$ + $CH_3Br$ reactants are set as the reference point.

| Nucleophile | Energy (eV) | Nucleophile | Energy (eV) |
|---|---|---|---|
| $HOO^{-}$ | 0 | $CH_3O^{-}$ | 5.8 |
| $HOO^{-}(CH_3OH)$ | -28.3 | $CH_3O^{-}(HOOH)$ | -32.3 |
| $HOO^{-}(CH_3OH)_2$ | -49.8 | $CH_3O^{-}(HOOH)(CH_3OH)$ | -54.4 |

Table S2. ADCH charge distributions of inv-$S_N2$-TS structures for $HOO^-(CH_3OH)_n$ + $CH_3Br$ and $CH_3O^-(HOOH)_{0,1}(CH_3OH)_{n-1}$ + $CH_3Br$ reactions.

| Nucleophile | $q$(O) | $q$(Nu) | $q$(C) | $q$($CH_3$) | $q$(Br) | $\Delta q$(Br–Nu) |
|---|---|---|---|---|---|---|
| $HOO^-$ | -0.538 | -0.784 | -0.075 | 0.297 | -0.513 | 0.271 |
| $HOO^-(CH_3OH)$ | -0.379 | -0.751 | -0.062 | 0.335 | -0.584 | 0.167 |
| $HOO^-(CH_3OH)_2$ | -0.157 | -0.686 | -0.081 | 0.300 | -0.614 | 0.072 |
| $CH_3O^-$ | -0.688 | -0.800 | -0.108 | 0.286 | -0.486 | 0.313 |
| $CH_3O^-(HOOH)$ | -0.414 | -0.690 | -0.108 | 0.291 | -0.600 | 0.090 |
| $CH_3O^-(HOOH)(CH_3OH)$ | -0.342 | -0.679 | -0.101 | 0.293 | -0.614 | 0.064 |

Table S3. Selected bond distances (Å) of inv-$S_N$2-TS structures for $HOO^{-}(CH_3OH)_n$ and $CH_3O^{-}(HOOH)_{0,1}(CH_3OH)_{n-1}$ reactions as optimized by MP2/ECP/d method.

| Nucleophile | $r^{TS}$(O–C) | $r^{TS}$(C–Br) | %(O–C) | %(C–Br) | %$L$ | %$AS$ |
|---|---|---|---|---|---|---|
| $HOO^{-}$ | 2.268 | 2.155 | 58.8 | 10.5 | 69.3 | 48.3 |
| $HOO^{-}(CH_3OH)$ | 2.109 | 2.258 | 47.7 | 15.8 | 63.5 | 31.9 |
| $HOO^{-}(CH_3OH)_2$ | 2.034 | 2.312 | 42.4 | 18.6 | 61.0 | 23.9 |
| $CH_3O^{-}$ | 2.314 | 2.126 | 62.6 | 9.0 | 71.6 | 53.6 |
| $CH_3O^{-}(HOOH)$ | 2.076 | 2.284 | 45.9 | 17.1 | 63.0 | 28.7 |
| $CH_3O^{-}(HOOH)(CH_3OH)$ | 2.041 | 2.313 | 43.4 | 18.6 | 62.0 | 24.8 |

Note: The calculated O−C bond length in $CH_3OOH$ is 1.428 Å, O−C bond length in $CH_3OCH_3$ is 1.423 Å, C−Br bond length in $CH_3Br$ is 1.950 Å.

Table S4. Energy (in eV) of the HOMO orbitals of the $HOO^{-}(CH_3OH)_n$ and $CH_3O^{-}(HOOH)_{0,1}(CH_3OH)_{n-1}$.

| Nucleophile | MP2/aug-cc-pVDZ | MP2/aug-cc-pVDZ (PCM) |
|---|---|---|
| $HOO^{-}$ | -3.19 | -9.16 |
| $HOO^{-}(CH_3OH)$ | -4.84 | -9.69 |
| $HOO^{-}(CH_3OH)_2$ | -5.72 | -10.06 |
| $CH_3O^{-}$ | -2.87 | -8.65 |
| $CH_3O^{-}(HOOH)$ | -5.02 | -9.74 |
| $CH_3O^{-}(HOOH)(CH_3OH)$ | -5.80 | -10.05 |
